# 2-Methyl­anilinium 3,4,5,6-tetra­bromo-2-(meth­oxy­carbon­yl)benzoate methanol monosolvate

**DOI:** 10.1107/S1600536811008543

**Published:** 2011-03-15

**Authors:** Jian Li

**Affiliations:** a Department of Chemistry and Chemical Engineering, Weifang University, Weifang 261061, People’s Republic of China

## Abstract

In the anion of the title compound, C_7_H_10_N^+^·C_9_H_3_Br_4_O_4_^−^·CH_3_O, the dihedral angles formed by the benzene ring and the mean planes of the carboxyl­ate and meth­oxy­carbonyl groups are 74.8 (5) and 75.0 (5)°, respectively. In the crystal, inter­molecular N—H⋯O and O—H⋯O hydrogen bonds link the components into chains along [100]. Additional stabilization is provided by weak inter­molecular C—H⋯O hydrogen bonds.

## Related literature

For related structures, see: Li (2011 ▶); Liang (2008 ▶). 
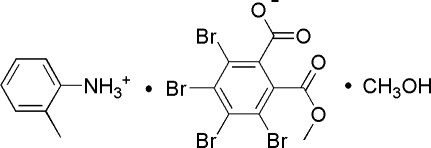


## Experimental

### 

#### Crystal data

C_7_H_10_N^+^·C_9_H_3_Br_4_O_4_^−^·CH_4_O
*M* *_r_* = 634.96
Monoclinic, 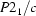

*a* = 8.1909 (8) Å
*b* = 13.5551 (12) Å
*c* = 19.5082 (16) Å
β = 90.371 (1)°
*V* = 2165.9 (3) Å^3^
*Z* = 4
Mo *K*α radiation
μ = 7.46 mm^−1^
*T* = 298 K
0.40 × 0.32 × 0.28 mm

#### Data collection

Bruker SMART CCD diffractometer
Absorption correction: multi-scan (*SADABS*; Bruker, 1997 ▶) *T*_min_ = 0.154, *T*_max_ = 0.229
10672 measured reflections
3811 independent reflections
2507 reflections with *I* > 2σ(*I*)
*R*_int_ = 0.055

#### Refinement

*R*[*F* ^2^ > 2σ(*F* ^2^)] = 0.037
*wR*(*F* ^2^) = 0.072
*S* = 1.07
3811 reflections
248 parameters
H-atom parameters constrained
Δρ_max_ = 0.71 e Å^−3^
Δρ_min_ = −0.59 e Å^−3^

### 

Data collection: *SMART* (Bruker, 1997 ▶); cell refinement: *SAINT* (Bruker, 1997 ▶); data reduction: *SAINT*; program(s) used to solve structure: *SHELXS97* (Sheldrick, 2008 ▶); program(s) used to refine structure: *SHELXL97* (Sheldrick, 2008 ▶); molecular graphics: *SHELXTL* (Sheldrick, 2008 ▶) and *PLATON* (Spek, 2009 ▶); software used to prepare material for publication: *SHELXTL*.

## Supplementary Material

Crystal structure: contains datablocks global, I. DOI: 10.1107/S1600536811008543/lh5212sup1.cif

Structure factors: contains datablocks I. DOI: 10.1107/S1600536811008543/lh5212Isup2.hkl

Additional supplementary materials:  crystallographic information; 3D view; checkCIF report

## Figures and Tables

**Table 1:** Hydrogen-bond geometry (Å, °)

*D*—H⋯*A*	*D*—H	H⋯*A*	*D*⋯*A*	*D*—H⋯*A*
N1—H1*A*⋯O5^i^	0.89	1.87	2.756 (6)	178
N1—H1*B*⋯O4^i^	0.89	1.87	2.746 (5)	170
O5—H5⋯O3^ii^	0.82	1.83	2.645 (5)	173
C15—H15⋯O5^i^	0.93	2.55	3.281 (7)	135
C17—H17*B*⋯O2	0.96	2.47	3.296 (9)	144

Symmetry codes: (i) 
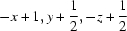
; (ii) 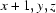.

**Fig. 1.**
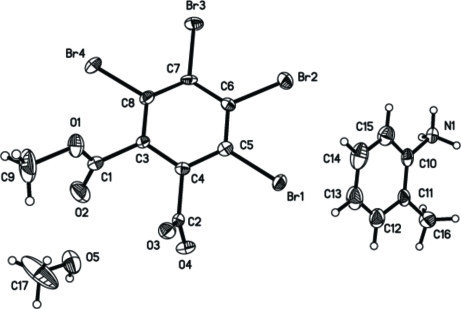
The molecular structure of (I), drawn with 30% probability ellipsoids.

**Fig. 2.**
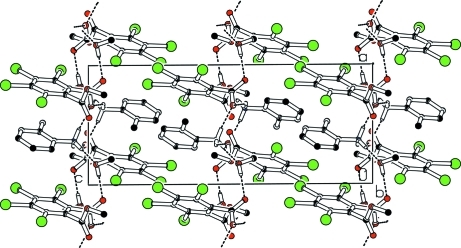
Part of the crystal structure of (I) with hydrogen bonds shown as dashed lines.

## supplementary crystallographic information

### Comment

4,5,6,7-Tetrabromo-2-ethylisoindoline-1,3-dione is an important flame retardant. 3,4,5,6-tetrabromo-2-(Methoxycarbonyl)benzoic acid is an intermediate in the sytnthesis of this flame retardant. In this paper, the structure of the title compound is reported. The asymmetric unit of the title compound (I) contains one *o*-toluidinium cation, one 3,4,5,6-tetrabromo-2-(methoxycarbonyl)benzoate anion and one methanol solvent molecule (Fig. 1). In the anion, the dihedral angles formed by the benzene ring and the mean-planes of the carboxylate and methoxycarbonyl groups are 74.8 (5) and 75.0 (5) °, respectively. The bond lengths and angles are in agreement with those which are related in ethylammonium 2-(methoxycarbonyl)-3,4,5,6-tetrabromobenzoate methanol solvate (Li, 2011) and in ethane-1,2-diammonium bis(2-(methoxycarbonyl)-3,4,5,6-tetrabromobenzoate) methanol solvate (Liang, 2008). In the crystal, intermolecular N—H···O and O—H···O hydrogen bonds link the components of the structure into one-dimensional chains along [100] (Fig. 2). Additional stabilization is provided by weak intermolecular C-H···O hydrogen bonds.

### Experimental

A mixture of 4,5,6,7-tetrabromoisobenzofuran-1,3-dione (4.64 g, 0.01 mol) and methanol (15 ml) was refluxed for 0.5 h and then *o*-toluidine (1.07 g, 0.01 mol) was added to the above solution. The solution was mixed 20 min at room temperature. This solution was kept at room temperature for 5 d. Natural evaporation gave colourless single crystals of the title compound, suitable for X-ray analysis.

### Refinement

H atoms were initially located from difference maps and then refined in a riding model with C—H = 0.93–0.96 Å, N—H = 0.89 Å, O—H = 0.82 Å and *U*_iso_(H) = 1.2*U*_eq_(C) or 1.5*U*_eq_(O, N, methyl C).

### Crystal data

**Table:**

C_7_H_10_N^+^·C_9_H_3_Br_4_O_4_^−^·CH_4_O	*F*(000) = 1224
*M**_r_* = 634.96	*D*_x_ = 1.947 Mg m^−^^3^
Monoclinic, *P*2_1_/*c*	Mo *K*α radiation, λ = 0.71073 Å
Hall symbol: -P 2ybc	Cell parameters from 2873 reflections
*a* = 8.1909 (8) Å	θ = 2.6–23.9°
*b* = 13.5551 (12) Å	µ = 7.46 mm^−^^1^
*c* = 19.5082 (16) Å	*T* = 298 K
β = 90.371 (1)°	Block, colorless
*V* = 2165.9 (3) Å^3^	0.40 × 0.32 × 0.28 mm
*Z* = 4	

### Data collection

**Table:**

Bruker SMART CCD diffractometer	3811 independent reflections
Radiation source: fine-focus sealed tube	2507 reflections with *I* > 2σ(*I*)
graphite	*R*_int_ = 0.055
φ and ω scans	θ_max_ = 25.0°, θ_min_ = 2.6°
Absorption correction: multi-scan (*SADABS*; Bruker, 1997)	*h* = −9→9
*T*_min_ = 0.154, *T*_max_ = 0.229	*k* = −13→16
10672 measured reflections	*l* = −23→21

### Refinement

**Table:**

Refinement on *F*^2^	Primary atom site location: structure-invariant direct methods
Least-squares matrix: full	Secondary atom site location: difference Fourier map
*R*[*F*^2^ > 2σ(*F*^2^)] = 0.037	Hydrogen site location: inferred from neighbouring sites
*wR*(*F*^2^) = 0.072	H-atom parameters constrained
*S* = 1.07	*w* = 1/[σ^2^(*F*_o_^2^) + (0.0232*P*)^2^] where *P* = (*F*_o_^2^ + 2*F*_c_^2^)/3
3811 reflections	(Δ/σ)_max_ < 0.001
248 parameters	Δρ_max_ = 0.71 e Å^−^^3^
0 restraints	Δρ_min_ = −0.59 e Å^−^^3^

### Special details

**Table:**

Geometry. All e.s.d.'s (except the e.s.d. in the dihedral angle between two l.s. planes) are estimated using the full covariance matrix. The cell e.s.d.'s are taken into account individually in the estimation of e.s.d.'s in distances, angles and torsion angles; correlations between e.s.d.'s in cell parameters are only used when they are defined by crystal symmetry. An approximate (isotropic) treatment of cell e.s.d.'s is used for estimating e.s.d.'s involving l.s. planes.
Refinement. Refinement of *F*^2^ against ALL reflections. The weighted *R*-factor *wR* and goodness of fit *S* are based on *F*^2^, conventional *R*-factors *R* are based on *F*, with *F* set to zero for negative *F*^2^. The threshold expression of *F*^2^ > σ(*F*^2^) is used only for calculating *R*-factors(gt) *etc*. and is not relevant to the choice of reflections for refinement. *R*-factors based on *F*^2^ are statistically about twice as large as those based on *F*, and *R*- factors based on ALL data will be even larger.

### Fractional atomic coordinates and isotropic or equivalent isotropic displacement parameters (Å^2^)

**Table:**

	*x*	*y*	*z*	*U*_iso_*/*U*_eq_	
Br1	0.03655 (7)	0.49170 (4)	0.40325 (2)	0.03923 (16)	
Br2	0.02019 (8)	0.40894 (4)	0.24560 (2)	0.05302 (19)	
Br3	0.15610 (7)	0.18668 (4)	0.20996 (2)	0.05158 (18)	
Br4	0.29944 (7)	0.04678 (4)	0.33723 (3)	0.05207 (19)	
N1	0.3657 (5)	0.8858 (3)	0.05060 (18)	0.0390 (11)	
H1A	0.3027	0.8481	0.0241	0.058*	
H1B	0.4659	0.8882	0.0333	0.058*	
H1C	0.3243	0.9464	0.0524	0.058*	
O1	0.1935 (5)	0.0866 (3)	0.49814 (18)	0.0577 (11)	
O2	0.4329 (5)	0.1646 (3)	0.5033 (2)	0.0712 (13)	
O3	0.1279 (4)	0.3275 (2)	0.55173 (15)	0.0374 (9)	
O4	0.3365 (4)	0.4141 (2)	0.51013 (15)	0.0386 (9)	
O5	0.8267 (5)	0.2644 (3)	0.5294 (2)	0.0714 (12)	
H5	0.9166	0.2887	0.5373	0.107*	
C1	0.3034 (8)	0.1514 (4)	0.4786 (2)	0.0398 (14)	
C2	0.2195 (6)	0.3546 (3)	0.5050 (2)	0.0268 (11)	
C3	0.2381 (5)	0.2138 (3)	0.4200 (2)	0.0265 (11)	
C4	0.1913 (5)	0.3107 (3)	0.4340 (2)	0.0242 (11)	
C5	0.1213 (6)	0.3663 (3)	0.3820 (2)	0.0293 (12)	
C6	0.1100 (6)	0.3289 (3)	0.3151 (2)	0.0297 (12)	
C7	0.1649 (6)	0.2341 (4)	0.3009 (2)	0.0315 (12)	
C8	0.2249 (6)	0.1765 (3)	0.3535 (2)	0.0326 (12)	
C9	0.2418 (9)	0.0245 (5)	0.5563 (3)	0.104 (3)	
H9A	0.3277	−0.0193	0.5425	0.156*	
H9B	0.1496	−0.0134	0.5712	0.156*	
H9C	0.2798	0.0654	0.5932	0.156*	
C10	0.3726 (6)	0.8439 (4)	0.1201 (3)	0.0410 (14)	
C11	0.4507 (7)	0.8936 (4)	0.1712 (3)	0.0450 (15)	
C12	0.4626 (8)	0.8472 (5)	0.2352 (3)	0.0645 (19)	
H12	0.5158	0.8790	0.2712	0.077*	
C13	0.3973 (9)	0.7565 (6)	0.2453 (3)	0.078 (2)	
H13	0.4090	0.7263	0.2878	0.094*	
C14	0.3140 (9)	0.7084 (5)	0.1937 (4)	0.080 (2)	
H14	0.2653	0.6475	0.2017	0.096*	
C15	0.3040 (7)	0.7521 (4)	0.1297 (3)	0.0584 (17)	
H15	0.2517	0.7199	0.0936	0.070*	
C16	0.5220 (7)	0.9958 (4)	0.1608 (3)	0.0619 (17)	
H16A	0.5957	0.9947	0.1227	0.093*	
H16B	0.5799	1.0156	0.2015	0.093*	
H16C	0.4354	1.0418	0.1516	0.093*	
C17	0.7840 (9)	0.2058 (7)	0.5817 (4)	0.159 (5)	
H17A	0.8389	0.1435	0.5776	0.238*	
H17B	0.6681	0.1954	0.5806	0.238*	
H17C	0.8145	0.2365	0.6242	0.238*	

### Atomic displacement parameters (Å^2^)

**Table:**

	*U*^11^	*U*^22^	*U*^33^	*U*^12^	*U*^13^	*U*^23^
Br1	0.0555 (4)	0.0286 (3)	0.0336 (3)	0.0129 (3)	0.0007 (2)	−0.0035 (2)
Br2	0.0803 (5)	0.0476 (4)	0.0309 (3)	0.0160 (3)	−0.0143 (3)	0.0012 (3)
Br3	0.0688 (4)	0.0524 (4)	0.0334 (3)	0.0099 (3)	−0.0113 (3)	−0.0209 (3)
Br4	0.0709 (5)	0.0284 (3)	0.0568 (4)	0.0109 (3)	−0.0097 (3)	−0.0151 (3)
N1	0.037 (3)	0.034 (3)	0.046 (3)	0.006 (2)	0.002 (2)	0.004 (2)
O1	0.076 (3)	0.041 (2)	0.056 (2)	−0.002 (2)	0.000 (2)	0.023 (2)
O2	0.066 (3)	0.076 (3)	0.071 (3)	0.000 (3)	−0.030 (2)	0.010 (2)
O3	0.046 (2)	0.037 (2)	0.0284 (18)	−0.0014 (17)	0.0035 (17)	0.0013 (16)
O4	0.042 (2)	0.038 (2)	0.0355 (19)	−0.0106 (19)	0.0016 (16)	−0.0121 (16)
O5	0.055 (3)	0.071 (3)	0.088 (3)	−0.016 (2)	−0.018 (2)	0.027 (3)
C1	0.053 (4)	0.030 (3)	0.036 (3)	0.004 (3)	−0.009 (3)	−0.009 (3)
C2	0.031 (3)	0.023 (3)	0.026 (3)	0.007 (3)	−0.004 (2)	−0.003 (2)
C3	0.029 (3)	0.022 (3)	0.029 (3)	−0.002 (2)	−0.005 (2)	−0.003 (2)
C4	0.023 (3)	0.026 (3)	0.023 (2)	−0.007 (2)	0.003 (2)	0.000 (2)
C5	0.029 (3)	0.029 (3)	0.029 (3)	−0.003 (2)	0.005 (2)	−0.001 (2)
C6	0.034 (3)	0.034 (3)	0.021 (2)	0.000 (2)	−0.003 (2)	0.000 (2)
C7	0.038 (3)	0.034 (3)	0.022 (2)	−0.004 (3)	−0.005 (2)	−0.013 (2)
C8	0.035 (3)	0.023 (3)	0.039 (3)	−0.003 (2)	−0.003 (2)	−0.005 (2)
C9	0.150 (8)	0.073 (5)	0.088 (5)	0.021 (5)	0.012 (5)	0.053 (4)
C10	0.036 (3)	0.044 (4)	0.043 (3)	0.015 (3)	0.012 (3)	0.009 (3)
C11	0.041 (4)	0.052 (4)	0.042 (3)	0.024 (3)	0.005 (3)	0.001 (3)
C12	0.081 (5)	0.066 (5)	0.047 (4)	0.033 (4)	0.008 (3)	0.000 (3)
C13	0.104 (6)	0.077 (6)	0.054 (4)	0.035 (5)	0.016 (4)	0.019 (4)
C14	0.085 (6)	0.055 (5)	0.100 (6)	0.006 (4)	0.031 (5)	0.031 (4)
C15	0.066 (5)	0.050 (4)	0.060 (4)	0.002 (3)	0.011 (3)	0.009 (3)
C16	0.061 (4)	0.065 (4)	0.060 (4)	0.010 (3)	−0.006 (3)	−0.011 (3)
C17	0.097 (7)	0.290 (13)	0.089 (6)	−0.103 (8)	−0.040 (5)	0.090 (7)

### Geometric parameters (Å, °)

**Table:**

Br1—C5	1.883 (5)	C7—C8	1.376 (6)
Br2—C6	1.883 (4)	C9—H9A	0.9600
Br3—C7	1.889 (4)	C9—H9B	0.9600
Br4—C8	1.889 (5)	C9—H9C	0.9600
N1—C10	1.471 (6)	C10—C11	1.359 (7)
N1—H1A	0.8900	C10—C15	1.379 (7)
N1—H1B	0.8900	C11—C12	1.402 (7)
N1—H1C	0.8900	C11—C16	1.518 (7)
O1—C1	1.315 (6)	C12—C13	1.356 (8)
O1—C9	1.464 (6)	C12—H12	0.9300
O2—C1	1.176 (6)	C13—C14	1.377 (9)
O3—C2	1.239 (5)	C13—H13	0.9300
O4—C2	1.256 (5)	C14—C15	1.383 (8)
O5—C17	1.341 (7)	C14—H14	0.9300
O5—H5	0.8200	C15—H15	0.9300
C1—C3	1.517 (6)	C16—H16A	0.9600
C2—C4	1.525 (6)	C16—H16B	0.9600
C3—C4	1.395 (6)	C16—H16C	0.9600
C3—C8	1.397 (6)	C17—H17A	0.9600
C4—C5	1.385 (6)	C17—H17B	0.9600
C5—C6	1.403 (6)	C17—H17C	0.9600
C6—C7	1.390 (6)		
			
C10—N1—H1A	109.5	H9A—C9—H9B	109.5
C10—N1—H1B	109.5	O1—C9—H9C	109.5
H1A—N1—H1B	109.5	H9A—C9—H9C	109.5
C10—N1—H1C	109.5	H9B—C9—H9C	109.5
H1A—N1—H1C	109.5	C11—C10—C15	122.6 (5)
H1B—N1—H1C	109.5	C11—C10—N1	120.0 (5)
C1—O1—C9	115.2 (5)	C15—C10—N1	117.4 (5)
C17—O5—H5	109.5	C10—C11—C12	117.4 (5)
O2—C1—O1	126.9 (5)	C10—C11—C16	122.3 (5)
O2—C1—C3	122.4 (5)	C12—C11—C16	120.3 (6)
O1—C1—C3	110.6 (5)	C13—C12—C11	120.7 (6)
O3—C2—O4	126.6 (4)	C13—C12—H12	119.7
O3—C2—C4	117.7 (4)	C11—C12—H12	119.7
O4—C2—C4	115.7 (4)	C12—C13—C14	121.2 (6)
C4—C3—C8	120.1 (4)	C12—C13—H13	119.4
C4—C3—C1	118.3 (4)	C14—C13—H13	119.4
C8—C3—C1	121.6 (4)	C13—C14—C15	118.9 (6)
C5—C4—C3	118.9 (4)	C13—C14—H14	120.6
C5—C4—C2	120.8 (4)	C15—C14—H14	120.6
C3—C4—C2	120.3 (4)	C10—C15—C14	119.2 (6)
C4—C5—C6	120.6 (4)	C10—C15—H15	120.4
C4—C5—Br1	118.8 (3)	C14—C15—H15	120.4
C6—C5—Br1	120.6 (3)	C11—C16—H16A	109.5
C7—C6—C5	120.0 (4)	C11—C16—H16B	109.5
C7—C6—Br2	121.0 (3)	H16A—C16—H16B	109.5
C5—C6—Br2	119.0 (3)	C11—C16—H16C	109.5
C8—C7—C6	119.4 (4)	H16A—C16—H16C	109.5
C8—C7—Br3	121.2 (4)	H16B—C16—H16C	109.5
C6—C7—Br3	119.4 (3)	O5—C17—H17A	109.5
C7—C8—C3	120.8 (4)	O5—C17—H17B	109.5
C7—C8—Br4	121.1 (3)	H17A—C17—H17B	109.5
C3—C8—Br4	118.0 (3)	O5—C17—H17C	109.5
O1—C9—H9A	109.5	H17A—C17—H17C	109.5
O1—C9—H9B	109.5	H17B—C17—H17C	109.5
			
C9—O1—C1—O2	−0.9 (8)	Br2—C6—C7—C8	−177.9 (4)
C9—O1—C1—C3	−178.0 (4)	C5—C6—C7—Br3	−177.7 (3)
O2—C1—C3—C4	−72.0 (7)	Br2—C6—C7—Br3	2.2 (6)
O1—C1—C3—C4	105.2 (5)	C6—C7—C8—C3	−2.9 (7)
O2—C1—C3—C8	107.7 (6)	Br3—C7—C8—C3	177.0 (3)
O1—C1—C3—C8	−75.1 (6)	C6—C7—C8—Br4	179.5 (3)
C8—C3—C4—C5	4.5 (7)	Br3—C7—C8—Br4	−0.6 (6)
C1—C3—C4—C5	−175.7 (4)	C4—C3—C8—C7	−0.5 (7)
C8—C3—C4—C2	−174.5 (4)	C1—C3—C8—C7	179.8 (5)
C1—C3—C4—C2	5.2 (7)	C4—C3—C8—Br4	177.2 (3)
O3—C2—C4—C5	106.2 (5)	C1—C3—C8—Br4	−2.5 (6)
O4—C2—C4—C5	−74.8 (6)	C15—C10—C11—C12	1.1 (8)
O3—C2—C4—C3	−74.8 (6)	N1—C10—C11—C12	−176.0 (4)
O4—C2—C4—C3	104.2 (5)	C15—C10—C11—C16	−178.3 (5)
C3—C4—C5—C6	−5.2 (7)	N1—C10—C11—C16	4.6 (8)
C2—C4—C5—C6	173.8 (4)	C10—C11—C12—C13	−0.4 (8)
C3—C4—C5—Br1	173.1 (3)	C16—C11—C12—C13	179.1 (6)
C2—C4—C5—Br1	−7.8 (6)	C11—C12—C13—C14	−1.7 (10)
C4—C5—C6—C7	1.9 (7)	C12—C13—C14—C15	3.0 (10)
Br1—C5—C6—C7	−176.4 (3)	C11—C10—C15—C14	0.2 (8)
C4—C5—C6—Br2	−178.0 (3)	N1—C10—C15—C14	177.4 (5)
Br1—C5—C6—Br2	3.7 (5)	C13—C14—C15—C10	−2.2 (9)
C5—C6—C7—C8	2.2 (7)		

### Hydrogen-bond geometry (Å, °)

**Table:**

*D*—H···*A*	*D*—H	H···*A*	*D*···*A*	*D*—H···*A*
N1—H1A···O5^i^	0.89	1.87	2.756 (6)	178
N1—H1B···O4^i^	0.89	1.87	2.746 (5)	170
O5—H5···O3^ii^	0.82	1.83	2.645 (5)	173
C15—H15···O5^i^	0.93	2.55	3.281 (7)	135
C17—H17B···O2	0.96	2.47	3.296 (9)	144

Symmetry codes: (i) −*x*+1, *y*+1/2, −*z*+1/2; (ii) *x*+1, *y*, *z*.
